# Expressed racial identity and hypertension in a telephone survey sample from Toronto and Vancouver, Canada: do socioeconomic status, perceived discrimination and psychosocial stress explain the relatively high risk of hypertension for Black Canadians?

**DOI:** 10.1186/1475-9276-11-58

**Published:** 2012-10-12

**Authors:** Gerry Veenstra

**Affiliations:** 1 Department of Sociology, The University of British Columbia, Vancouver, British Columbia, Canada

**Keywords:** Canada, Racial identity, Hypertension, Socioeconomic status, Perceived discrimination, Psychosocial stress

## Abstract

**Introduction:**

Canadian research on racial health inequalities that foregrounds socially constructed racial identities and social factors which can explain consequent racial health inequalities is rare. This paper adopts a social typology of salient racial identities in contemporary Canada, empirically documents consequent racial inequalities in hypertension in an original survey dataset from Toronto and Vancouver, Canada, and then attempts to explain the inequalities in hypertension with information on socioeconomic status, perceived experiences with institutionalized and interpersonal discrimination, and psychosocial stress.

**Methods:**

Telephone interviews were conducted in 2009 with 706 randomly selected adults living in the City of Toronto and 838 randomly selected adults living in the Vancouver Census Metropolitan Area. Bivariate analyses and logistic regression modeling were used to examine relationships between racial identity, hypertension, socio-demographic factors, socioeconomic status, perceived discrimination and psychosocial stress.

**Results:**

The Black Canadians in the sample were the most likely to report major and routine discriminatory experiences and were the least educated and the poorest. Black respondents were significantly more likely than Asian, South Asian and White respondents to report hypertension controlling for age, immigrant status and city of residence. Of the explanatory factors examined in this study, only educational attainment explained some of the relative risk of hypertension for Black respondents. Most of the risk remained unexplained in the models.

**Conclusions:**

Consistent with previous Canadian research, socioeconomic status explained a small portion of the relatively high risk of hypertension documented for the Black respondents. Perceived experiences of discrimination both major and routine and self-reported psychosocial stress did not explain these racial inequalities in hypertension. Conducting subgroup analyses by gender, discerning between real and perceived experiences of discrimination and considering potentially moderating factors such as coping strategy and internalization of racial stereotypes are important issues to address in future Canadian racial inequalities research of this kind.

## Introduction

Hypertension is one of the most common chronic diseases in modern-day western societies. In Canada, more than one in five adults lives with the disease [1]. In the United States, almost one-third of adults aged 20 and over is hypertensive [2]. A large body of research has established the existence of sizeable racial inequalities in hypertension in the latter nation, one notable facet of which is the elevated risk of hypertension for African Americans compared to White Americans. Data from the U.S. National Health and Nutrition Examination Survey (NHANES) reveal rates of hypertension in 1988–1994 that are 1.4 times as high among Black men than among White men and 1.8 times as high among Black women than among White women [3]. More recent studies have determined that the Black-White gap in hypertension in the United States is not shrinking and may even be widening [4].

Various explanations have been proposed for the persistent Black-White gap in hypertension in the United States, including the residential segregation of racial minorities, differences in socioeconomic status, differential access to quality health care, internalization by racial minorities of the larger society’s negative characterizations of them, and the psychosocial stress that can result from experiences of interpersonal racism and discrimination [3]. All of these explanations have received empirical attention from American researchers, albeit to different degrees. The mediating role played by socioeconomic status in Black-White differences in hypertension has been the most extensively investigated. Many researchers have found that socioeconomic status explains some of the Black-White gap in hypertension [5,6]. It does not, however, explain the entirety of the gap [7,8], inviting other explanations. Subjective perceptions regarding experiences of discrimination have received attention in recent years as another plausible explanation for Black-White differences in hypertension in the United States. Researchers posit that chronic exposure to perceived interpersonal discrimination induces a psychosocial stress which in turn elevates blood pressure. A review of the literature on perceived racism and blood pressure finds that the American evidence linking perceived racism and discrimination to blood pressure in general is mixed [9]. Most of the relevant studies examine linkages between perceived discrimination and blood pressure within rather than between racial groupings. One notable study, however, examined the degree to which perceived discrimination explained Black-White differences in hypertension [10]: it found that Black-White differences were wider among working-class women reporting some discrimination than among working-class women reporting none, a result that runs contrary to the psychosocial stress hypothesis. Scholars have posited that perceived racism may only adversely affect blood pressure in certain conditions, e.g., when the people perceiving the discrimination are disposed to actively cope with the discrimination and overcome adversity [11] or when they do not internalize negative stereotypes about their own racial group [12]. Research in this complex field of inquiry continues.

In contrast with the laudable efforts of the American health research community, racial inequalities in hypertension in Canada have yet to receive sustained attention from health researchers. This may reflect reluctance on the part of the Canadian health research community to countenance the possibility of health inequalities by ‘race’ in this supposedly egalitarian, multicultural and tolerant society. But Canada is not egalitarian or tolerant, and racial health inequalities do in fact exist here, as documented in a small number of empirical studies [13-20]. With regard to hypertension in particular, one of the studies reported an age-adjusted prevalence of hypertension of 17.1% for White Canadians compared to an age-adjusted prevalence of 23.9% for Black Canadians [19]. Another nationally representative study found that Black Canadians had odds of reporting hypertension that were 1.56 times as high as those of White Canadians controlling for age and gender, a relationship that was not explained by socioeconomic status or urban versus rural locale [15]. Overall, however, little is currently known about the nature of racial differences in hypertension in Canada and, to the degree that they exist, the viability of the different possible explanations for them.

Accordingly, this paper adopts a social typology of salient racial identities in contemporary Canada, empirically documents racial inequalities in hypertension in an original survey dataset from Toronto and Vancouver, Canada, and then attempts to explain the inequalities with information on socioeconomic status, perceived experiences with institutionalized and interpersonal discrimination, and psychosocial stress. It extends to Canada a line of inquiry already well established in the United States and elsewhere, thereby contributing to the illumination of racial inequalities in hypertension in Canada and the viability of several proposed explanations for them.

## Methods

### Survey sample

In 2009, the Survey Research Centre (SRC) at the University of Victoria conducted telephone interviews with 732 adults living in the City of Toronto and 863 adults living in the Vancouver Census Metropolitan Area. The survey was approved by the Research Ethics Board at the University of British Columbia. The SRC used random-digit dialing techniques to obtain residential telephone numbers, a next-birthday strategy to select one resident per household aged 19 or older to interview and a computer-aided telephone interviewing system to conduct the interviews. The racial identities of the interviewers were not recorded. The cooperation rate was 9.3%. A total of 1,544 respondents (96.8% of the full sample) had non-missing information for all of hypertension, age, gender, marital status, city of residence, immigrant status, educational attainment and psychosocial stress. Characteristics of this working sample are described in Table 1. Comparing the city samples to the 2006 Census by age, gender, marital status, immigrant status and education, both city samples were biased towards older people, women, non-immigrants and university-educated people. In addition, the Toronto sample was biased towards unmarried people and the Vancouver sample was biased towards married people.

**Table 1:** **Characteristics of the sample (*n*=1,544)**

**Variable**		**City of residence**	
		**Toronto**	**Vancouver**
		**n(%)**	**n(%)**
Expressed racial identity	Aboriginal	3(0.4)	4(0.5)
	Asian	20(2.8)	50(6.0)
	Black	45(6.4)	2(0.2)
	South Asian	26(3.7)	42(5.0)
	Southeast Asian	10(1.4)	10(1.2)
	White	539(76.4)	683(81.5)
	Other	16(2.3)	19(2.3)
	multiple responses	20(2.8)	12(1.4)
	missing	27(3.8)	16(1.9)
Age	aged 19-34	144(20.4)	104(12.4)
	aged 35-44	133(18.8)	137(16.4)
	aged 45 - 54	163(23.1)	199(23.8)
	aged 55-64	141(20.0)	255(26.9)
	aged 65 and other	125(17.7)	173(20.6)
Gender	male	251(35.6)	274(32.7)
	female	455(64.4)	564(67.3)
Marital status	married or common law	368(52.1)	522(62.3)
	separated, divorced or widowed	161(22.8)	189(22.6)
	never been married	177(25.1)	127(15.2)
Immigrant status	born in Canada	457(64.7)	608(72.6)
	immigrated > 20 years ago	169(23.9)	152(18.1)
	immigrated between 10 and 20 years ago	39(5.5)	41(4.9)
	immigrated < 10 years ago	41(5.8)	37(4.4)
Educational attainment	high school or less	157(22.2)	235(28.0)
	CC, TS or some university	160(22.7)	257(30.7)
	bachelor’s degree	237(33.6)	216(25.8)
	post - graduate degree	152(21.5)	130(15.5)
Household income	less than $40,000	110(15.6)	130(15.5)
	$40,000 - $59,999	89(12.6)	98(11.7)
	$60,000 - $79,999	103(14.6)	99(11.7)
	$80,000 - $99,000	72(10.2)	89(10.6)
	$100,000 - $149,999	101(14.3)	151(18.0)
	$150,000 or more	124(17.6)	121(14.4)
	missing	107(15.2)	150(17.9)
Major discrimination	no experiences	276(39.1)	384(45.8)
	one experience	194(17.5)	201(24.0)
	two experiences	106(15.0)	116(13.8)
	three or more experiences	88(12.5)	105(12.5)
	missing	42(6.0)	32(3.8)
Racial/ethnic major discriminatory experiences	no experiences	588(83.3)	735(87.7)
	one experience	55(7.8)	57(6.8)
	two or more experiences	21(3.0)	14(1.7)
	missing	42(6.0)	32(3.8)
Routine discrimination index	0	148(21.0)	226(27.0)
	1 or 2	194(27.5)	250(29.8)
	3 or 4	131(18.6)	147(17.5)
	5 or greater	205(29.0)	189(22.6)
	missing	28(4.0)	26(3.1)
Daily stress	not stressful	99(14.0)	161(19.2)
	a bit stressful	367(52.0)	437(52.2)
	quite or extremely stressful	240(34.0)	240(28.6)

### Survey measures

‘Expressed racial identity’ refers to a person’s self-identification with a racial grouping that s/he will express to others when asked to fit into ‘official’ racial classifications presented by Census forms, survey researchers, insurance forms, and so forth [16,21]. To measure expressed racial identity interviewees were asked: “Now I’d like to ask you about your racial background. How would you describe your racial background? For example, are you White, Asian, South Asian, Black, Southeast Asian, or Aboriginal, or perhaps something else I haven’t mentioned? Please feel free to provide more than one answer if you have several backgrounds.” Due to the small number of respondents who chose Aboriginal or Southeast Asian identity (Table 1), the expressed racial identity variable was subsequently recoded as Asian, Black, South Asian, White, and Other for these analyses.

Interviewers asked fifteen questions pertaining to experiences of discrimination. The items were adapted from the Major Experiences of Discrimination scale and the Everyday Discrimination Scale created by Williams and colleagues [22] and utilized by numerous others e.g., [23-25]. The items are intended to measure major experiences of unfair treatment as well as chronic, routine experiences of unfair treatment in everyday life. The preamble to the questions went as follows: “Now let’s go in a different direction. The following questions we will ask are personal in nature and may make you feel uncomfortable. Your responses will be kept in the strictest of confidence. We are interested in your opinions about how other people have treated you. Can you tell me if any of the following has ever happened to you?” Respondents were asked seven questions pertaining to major experiences of unfair treatment: 1. “For unfair reasons, have you ever not been hired for a job?” 2. “Have you ever been unfairly denied a promotion at work?” 3.” Do you feel you have been unfairly fired or let go from a job?” 4. “Have you ever moved into a neighborhood where neighbors made life difficult for you or your family?” 5. “Have you ever received service from someone such as a plumber or car mechanic that was worse than what other people get?” 6. “Have you ever been unfairly denied a bank loan, a mortgage, or insurance?” 7. “Have you ever been unfairly questioned, searched, or threatened by the police?” An alpha of 0.516 indicates that these dichotomous items did not form an internally coherent scale. An index of major discriminatory experiences was created that distinguished between three or more, two, one and no major experiences.

Each time a respondent responded in the affirmative to a major discriminatory experience question they were then asked “What do you think was the main reason for this experience?” The following possible responses were read aloud by interviewers in random order: Your gender, your age, your ethnic or racial background, your height, your weight, your religion, your education, your income level, a physical disability, your sexual orientation, other. From this a measure of racial/ethnic major discriminatory experiences in particular was created that distinguished between two or more experiences, one experience and no experiences of discrimination attributed by respondents to their racial/ethnic identities.

Respondents were also asked: “In your day-to-day life, how often do the following things happen to you?” 1. “You are treated with less respect or courtesy than other people.” 2. “You receive poorer service than other people at restaurants or stores.” 3. “People act as if they think you are not smart.” 4. “People act as if they think you are dishonest.” 5. “People act as if they think they are better than you.” 6. “People act as if they are afraid of you.” 7. “You are called names or insulted by people.” and 8. “You are threatened or harassed by people.” Possible responses to these questions were: almost every day, at least once a week, a few times a month, a few times a year, and never. An index of self-reported everyday discrimination was created by summing the responses to these questions (0 = never, 1 = a few times a year, 2 = a few times a month, 3 = at least once a week and 4 = almost every day) into a variable ranging from 0 to 22 with a mean of 3.205 and a standard deviation of 3.595 (*n* = 1490). An alpha of 0.794 indicates that respondents who experienced one form of routine discrimination were relatively likely to have experienced others as well, perhaps indicative of a latent factor pertaining to susceptibility to routine forms of discrimination more generally. This variable was subsequently recoded into categorical form as shown in Table 1.

Respondents were asked “What is the highest level of education you have completed?” with response categories that ranged from less than high school to a completed postgraduate degree. This variable was coded to distinguish between high school or less; community college, technical school or some university; bachelor’s degree at university; and postgraduate degree. To measure income, respondents were asked: “What is your best estimate of the total income of all household members – including yourself – in the year 2008, before taxes and deductions? Please be sure to include income from all sources.” A set of income ranges provided to respondents culminated in an upper category representing household incomes of $150,000 or higher (see Table 1).

To measure psychosocial stress, respondents were asked “Thinking about the amount of stress in your life, would you say that most days are: not at all stressful? a bit stressful? quite stressful? extremely stressful?” The latter two categories were combined due to small cell sizes.

Finally, respondents were asked: “Now I’d like to ask you about certain health conditions you may have. Do you have high blood pressure or hypertension?” If yes, they were asked: “Was your high blood pressure diagnosed by a physician?” Three hundred and six (19.8%) respondents reported hypertension, in all but seven cases diagnosed as such by a physician.

### Statistical analysis

The statistical analyses were conducted in Stata 11.2. Missing data categories were created for household income (*n* = 257), major experiences of discrimination (*n* = 74) and routine experiences of discrimination (*n* = 54). Bivariate relationships were investigated via comparisons of means with one-way ANOVA tests of significance and cross-tabulations with Chi-square tests of significance. Multivariate analyses involved binary logistic regression modeling of the presence of hypertension. The survey functionality of Stata was used to properly account for the complex survey design – two cities of different population sizes; different numbers of households randomly sampled within the two cities; sampling without replacement rather than with replacement when sampling households; one adult randomly selected from each household – in the regression modeling. This ensured that the correct sampling weights were used when generating point estimates and that the weighting, clustering and stratification characteristics of the survey design were properly accommodated when generating standard errors.

## Results

### Preliminary analyses

Table 2 describes bivariate relationships between socio-demographic characteristics, socioeconomic status, self-reported discrimination and psychosocial stress and the primary independent (expressed racial identity) and dependent (hypertension) variables. With regard to socio-demographic characteristics, expressed racial identity was significantly related to age (*F* = 35.24, *df* = 4, 1539, *p* < 0.001), with Black respondents the youngest and White respondents the oldest, on average; gender (Chi-square = 18.68, *df* = 4, *p* < 0.01), with the lowest proportion of women among South Asian respondents and the highest among Black respondents; marital status (Chi-square = 63.83, *df* = 8, *p* < 0.001), with South Asian respondents most likely and Black respondents least likely to be married or common-law; immigrant status (Chi-square = 388.20, *df* = 12, *p* < 0.001), with White respondents much more likely than others to be born in Canada and South Asian respondents most likely to be recent immigrants; city of residence (Chi-square = 63.76, *df* = 4, *p* < 0.001), with nearly all Black respondents living in Toronto and most Asian respondents living in Vancouver; education (Chi-square = 33.56, *df* = 12, *p* < 0.01), with Black respondents least and Asian respondents most educated; and income (Chi-square = 63.69, *df* = 24, *p* < 0.001), with Black respondents the poorest and White respondents the wealthiest. With regard to perceived discrimination, expressed racial identity was significantly related to major discriminatory experiences (Chi-square = 37.77, *df* = 16, *p* < 0.01), major racial/ethnic discriminatory experiences (Chi-square = 202.10, *df* = 12, *p* < 0.001) and routine discriminatory experiences (Chi-square = 82.29, *df* = 20, *p* < 0.001), with Black respondents reporting the most discriminatory experiences of all kinds, Asian and White respondents reporting the fewest major discriminatory experiences and White respondents reporting the fewest routine discriminatory experiences.

**Table 2:** Correlates of expressed racial identity and hypertension

**Variable**		**Expressed racial identity**					**Hypertension**	
		***Asian***	***Black***	***South Asian***	***White***	***Other***	***yes***	***no***
Age	in years - n (sd)	42.5(14.3)	40.5(11.2)	41.1(14.1)	53.6(14.8)	44.6(15.2)	61.75(12.5)	48.7(14.8)
		*F = 35.24, df = 4,1539, p < 0.001*					*F = 200.43, df = 1, 1542, p < 0.001*	
Gender	female n (%)	50(71.4)	37(78.7)	31(45.6)	817(66.9)	84(61.3)	196(19.2)	823(80.8)
	male	20(28.6)	10(21.3)	37(54.4)	405(33.1)	53(38.7)	110(20.9)	415(79.1)
		*Chi - square = 18.68, df = 4, p < 0.01*					*Chi - square = 0.64, df = 1, p > 0.10*	
Marital status	married or common law	32(45.7)	16(34.0)	47(69.1)	716(58.6)	79(57.7)	157(17.6)	733(82.4)
	separated, divorced or widowed	10(14.3)	9(19.2)	9(13.2)	303(24.8)	19(13.9)	114(32.6)	236(67.4)
	never been married	28(40.0)	22(46.8)	12(17.7)	203(16.6)	39(28.5)	35(11.5)	269(88.5)
		*Chi - square = 63.83, df = 8, p < 0.001*					*Chi - square = 51.67, df = 2, p < 0.001*	
Immigrant status	born in Canada	17(24.3)	12(25.5)	15(22.1)	962(78.7)	59(43.1)	211(19.8)	854(80.2)
	immigrated > 20 years ago	23(32.9)	24(51.1)	21(30.9)	213(17.4)	40(29.2)	81(25.2)	240(74.8)
	immigrated between 10 and 20 years ago	16(22.9)	6(12.8)	15(22.1)	22(1.8)	21(15.3)	8(10.0)	72(90.0)
	immigrated < 10 years ago	14(20.0)	5(10.6)	17(25.0)	25(2.1)	17(12.4)	6(7.7)	72(92.3)
		*Chi - square = 388.20, df = 12, p < 0.001*					*Chi - square = 17.99, df = 3, p < 0.001*	
City of residence	Toronto	20(28.6)	45(95.7)	26(38.2)	539(44.1)	76(55.5)	123(17.4)	583(82.6)
	Vancouver	50(71.4)	2(4.3)	42(61.8)	683(55.9)	61(44.5)	183(21.8)	655(78.2)
		*Chi - square = 63.76, df = 4, p < 0.001*					*Chi - square = 4.70, df = 1, p < 0.05*	
Educational attainment	high school or less	11(15.7)	18(38.3)	16(23.5)	318(26.0)	29(21.2)	95(24.3)	297(75.8)
	CC, TS or some university	12(17.1)	16(34.0)	15(22.1)	328(26.8)	46(33.6)	93(22.3)	324(77.7)
	bachelor’s degree	35(50.0)	12(25.5)	21(30.9)	343(28.1)	42(30.7)	76(16.8)	377(83.2)
	post - graduate degree	12(17.1)	1(2.1)	16(23.5)	233(19.1)	20(14.6)	42(14.9)	240(85.1)
		*Chi - square = 33.56, df = 12, p < 0.01*					*Chi - square = 13.37, df = 3, p < 0.01*	
Household income	less than $40,000	9(12.9)	17(36.2)	8(11.8)	181(14.8)	25(18.3)	58(24.2)	182(75.8)
	$40,000 - $59,999	15(21.4)	6(12.8)	10(14.7)	144(11.8)	12(8.8)	41(21.9)	146(78.1)
	$60,000 - $79,999	8(11.4)	6(12.8)	8(11.8)	158(12.9)	22(16.1)	33(16.3)	169(83.7)
	$80,000 - $99,000	7(10.0)	3(6.4)	10(14.7)	128(10.5)	13(9.5)	26(16.2)	135(83.9)
	$100,000 - 149,999	8(11.4)	5(10.6)	8(11.8)	216(17.1)	15(11.0)	50(19.8)	202(80.2)
	$150,000 or more	4(5.7)	1(2.1)	8(11.8)	217(17.8)	15(11.0)	43(17.6)	202(78.6)
	missing	19(27.1)	9(19.2)	16(23.5)	178(14.6)	35(25.6)	55(21.4)	202(78.6)
		*Chi -square = 63.69, df = 24, p < 0.001*					*Chi - square = 7.48, df = 6, p > 0.10*	
Major discrimination	no experiences	37(52.9)	14(29.8)	30(44.1)	534(43.7)	45(32.9)	130(19.7)	530(80.3)
	one experience	19(27.1)	7(14.9)	15(22.1)	315(25.8)	39(28.5)	85(21.5)	310(78.5)
	two experiences	7(10.0)	9(19.2)	9(13.2)	177(14.5)	20(14.6)	44(19.8)	178(80.2)
	three or more experiences	2(2.9)	10(21.3)	10(14.7)	149(12.2)	22(16.1)	37(19.2)	156(80.8)
	missing	5(7.1)	7(14.9)	4(5.9)	47(3.9)	11(8.0)	10(13.5)	64(86.5)
		*Chi - square = 37.77, df = 16, p < 0.01*					*Chi - square = 2.63, df = 4, p > 0.10*	
Racial/ethnic major discriminatory experiences	no experiences	51(72.9)	21(44.7)	43(63.2)	1113(91.1)	95(69.3)	274(20.7)	1049(79.3)
	one experience	9(12.9)	12(25.5)	13(19.1)	57(4.7)	21(15.3)	16(14.3)	96(85.7)
	two or more experiences	5(7.1)	7(14.9)	8(11.8)	5(0.4)	10(7.3)	6(17.1)	29(82.9)
	missing	4(7.1)	7(14.9)	4(5.9)	47(3.9)	11(8.0)	10(13.5)	64(86.5)
		*Chi - square = 202.10, df = 12, p < 0.001*					*Chi - square = 4.83, df = 3, p > 0.10*	
Routine discrimination index	0	14(20.0)	2(4.3)	13(19.1)	316(25.9)	29(21.2)	79(21.1)	295(78.9)
	1 or 2	18(25.7)	6(12.8)	24(35.3)	368(30.1)	28(20.4)	97(21.9)	347(78.2)
	3 or 4	10(14.3)	11(23.4)	7(10.3)	233(19.1)	17(12.4)	56(20.1)	222(79.9)
	5 or greater	22(31.4)	24(51.1)	21(30.9)	278(22.8)	49(35.8)	58(14.7)	336(85.3)
	missing	6(8.6)	4(8.5)	3(4.4)	27(2.2)	14(10.2)	16(29.6)	38(70.4)
		*Chi - square = 82.29, df = 20, p < 0.001*					*Chi - square = 11.28, df = 4, p < 0.05*	
Daily stress	not stressful	10(14.3)	8(17.0)	16(23.5)	201(16.5)	25(18.3)	65(25.0)	195(75.0)
	a bit stressful	44(62.9)	26(55.3)	35(51.5)	626(51.2)	73(53.3)	148(18.4)	656(81.6)
	quite or extremely stressful	16(22.9)	13(27.7)	17(25.0)	395(32.3)	39(28.5)	93(19.4)	387(80.6)
		*Chi - square = 7.64, df = 8, p > 0.10*					*Chi - square = 5.46, df = 2, p > 0.05*	

With regard to risk of hypertension and socio-demographic characteristics, there was a strong exponential effect of age on the likelihood of reporting hypertension (comparison of mean ages produced *F* = 200.43, *df* = 1, 1542, *p* < 0.001). Hypertension was also significantly related to marital status (Chi-square = 51.67, *df* = 2, *p* < 0.001), with respondents who had never been married the least likely to report hypertension, a result that is a function of age (results not shown); immigrant status (Chi-square = 17.99, *df* = 3, *p* < 0.001), where, consistent with the healthy immigrant effect, recent immigrants were the least likely to report hypertension and longstanding immigrants were the most likely to do so; city of residence (Chi-square = 4.70, *df* = 1, *p* < 0.05), with Vancouver residents more likely than Toronto residents to report hypertension; and education (Chi-square = 13.37, *df* = 3, *p* < 0.01), with better-educated respondents the less likely to report hypertension. With regard to perceived discrimination, the major discriminatory experiences (Chi-square = 2.63, *df* = 4, *p* > 0.10), racial/ethnic experiences of discrimination (Chi-square = 4.83, *df* = 3, *p* > 0.10) and psychosocial stress (Chi-square = 5.46, *df* = 2, *p* > 0.05) variables were not significantly related to hypertension. Surprisingly, routine discriminatory experiences were related to hypertension in the unexpected direction wherein more perceived discrimination of this kind corresponded with a lower likelihood of reporting high blood pressure (Chi-square = 11.28, *df* = 4, *p* < 0.05).

Findings from these preliminary analyses indicate the importance of controlling for age, immigrant status and city of residence in an investigation of relationships between expressed racial identity and hypertension. They also speak to the plausibility of educational attainment and routine discriminatory experiences, and the implausibility of household income, major experiences of discrimination, racial/ethnic discrimination, and psychosocial stress, as mediating factors in causal pathways from expressed racial identity to hypertension in this sample.

### Modeling hypertension

Table 3 describes the results from a series of three binary logistic regression models performed on hypertension. The first model controls for age, immigrant status and city of residence, the second model additionally controls for educational attainment and the third model adds the routine discrimination variable to the second model.

**Table 3:** Binary logistic regression models on hypertension

		**Model 1**		**Model 2**		**Model 3**	
		***OR***	***95% Cl***	***OR***	***95% Cl***	***OR***	***95% Cl***
Expressed racial identity	Asian	0.95	0.41 . 2.21	0.95	0.40 . 2.27	0.94	0.39 . 2.29
	Black	5.16***	2.26 . 11.77	4.42**	1.90 . 10.29	4.22**	1.80 . 9.88
	South Asian	0.58	0.22 . 1.54	0.59	0.21 . 1.64	0.59	0.21 . 1.62
	Other	1.77	0.95 . 3.31	1.74	0.93 . 3.26	1.74	0.91 . 3.31
	White(reference)	1.00		1.00		1.00	
Age in years	-----	1.26***	1.16 . 1.37	1.26***	1.16 . 1.37	1.26***	1.16 . 1.38
Age in years squared	-----	1.00***	1.00 . 1.00	1.00***	1.00 . 1.00	1.00***	1.00 . 1.00
Immigration status	immigrated > 20 years ago	1.05	0.72 . 1.55	1.11	0.75 . 1.63	1.11	0.75 . 1.64
	immigrated 10–19 years ago	1.44	0.53 . 3.91	1.54	0.55 . 4.28	1.51	0.54 . 4.23
	immigrated < 10 years ago	1.42	0.56 . 3.58	1.70	0.67 . 4.36	1.74	0.68 . 4.43
	born in Canada(reference)	1.00		1.00		1.00	
City of residence	Vancouver	1.23	0.90 . 1.68	1.17	0.85 . 1.60	1.18	0.85 . 1.63
Education	high school or less	-----	-----	0.80	0.53 . 1.20	2.01**	1.24 . 3.27
	c c/ts/come university	-----	-----	0.64*	0.42 . 0.98	1.59	0.98 . 2.58
	bachelor degree	-----	-----	0.50**	0.31 . 0.81	1.29	0.79 . 2.11
	post-graduate degree (reference)	-----	-----	1.00		1.00	
Routine discrimination scale	1 or 2	-----	-----	-----	-----	1.10	0.73 . 1.67
	3 or 4	-----	-----	-----	-----	1.28	0.78 . 2.08
	5 or greater	-----	-----	-----	-----	1.24	0.76 . 2.03
	missing	-----	-----	-----	-----	1.17	0.54 . 2.54
	0 (reference)	-----	-----	-----	-----	1.00	
N		1,544		1,544		1,544	
f test (*F*, df, p)		F = 13.21, df = 10, 1533, p < 0.001		F = 10.68, df = 13, 1530, p < 0.001		F = 8.20, df = 17, 1526, p < 0.001	
MacFadden pseudo R - squared		0.142		0.146		0.149	

Ten of 70 (14.3%) Asian respondents, 12 of 47 (25.5%) Black respondents, 6 of 68 (8.8%) South Asian respondents and 253 of 1,222 (20.7%) White respondents reported hypertension. Controlling for age, marital status, immigrant status and city of residence in Model 1, Black respondents were significantly more likely than White respondents (OR = 5.16, 95% CI = 2.26 … 11.77, *p* < 0.001) to report hypertension. (Black Canadian respondents were also significantly more likely than Asian Canadian respondents (OR = 5.41, 95% CI = 1.86 … 15.70, *p* < 0.01) and South Asian Canadian respondents (OR = 8.85, 95% CI = 2.80 … 27.93, *p* < 0.001) to report hypertension when Asian and South Asian, respectively, replaced White as the reference category.) The Black-White odds ratio was marginally attenuated upon additionally controlling for education (OR = 4.42, 95% CI = 1.90 … 10.29, *p* < 0.01). Additionally controlling for routine discriminatory experiences did not substantially reduce the effect (OR = 4.22, 95% CI = 1.80 … 9.88, *p* < 0.01).

In summary, Black respondents manifested relatively high risks of hypertension compared with Asian, South Asian and White respondents. Of the possible explanatory factors examined in this study, only educational attainment explained some of the risk of hypertension for Black respondents relative to the others. Most of the risk remained unexplained in these analyses.

## Discussion

### Limitations

Limitations of the study include the use of self-reported measures of health and a crude measure of self-reported psychosocial stress. The racial identity labels assessed in the study may not perfectly reflect current processes of racialization in this country; careful examination of Canadian media and interviews with a multitude of Canadians from all walks of life are needed in order to ascertain the terms and concepts that Canadians tend to use to map the fluid, ever-changing racial landscape in contemporary Canadian society [16]. Also, as in most survey research of this kind, the racial identity labels were attached to survey respondents on the basis of their own perceptions and affiliations. This means that, to the degree that key racialized identities in Canadian society were misidentified and/or imputed racialized identities and self-professed racial identities are incongruent with one another, serious measurement error exists. It is also worth noting that the measures of discrimination did not assess age of first onset and addressed a limited set of domains [3]. The cooperation rate was low bringing issues of representativeness into play. Another limitation of the study pertained to the small numbers of self-expressed Black respondents (*n* = 47) and self-expressed Asian respondents (*n* = 68) which limited the number of independent variables in the multivariate models and prevented subgroup analyses by gender or immigrant status. The exceedingly small numbers of Aboriginal (*n* = 7) and Southeast Asian (*n* = 20) respondents prevented any investigation of their relative risks of reporting hypertension, an especially important limitation given recent Canadian research which has identified relatively high risks of hypertension accruing to these identities [15].

### Study findings

The Black Canadians in the sample were the most likely to report major and routine discriminatory experiences and were the least educated and the poorest. These results, evidence of multiple kinds of systematic racism regularly encountered by Black Canadians, are consistent with previous research in Canada. For example, other studies have also reported that Black Canadians earn significantly less than White Canadians [26,27] and are the racial group most likely to report discrimination [28].

A relatively high risk of hypertension for Black Canadians also emerged in these data: controlling for age, immigrant status and city of residence, the odds of a Black respondent reporting hypertension were more than five times as high as those for Asian, South Asian and White respondents. The relatively high risk of hypertension among the Black respondents compared to the White respondents is consistent with previous research in the United States [3,9-11,29,30] and Canada [15,19].

Regarding potentially explanatory factors, the risk of hypertension for the Black Canadians was partly explained by education; none of the other factors meaningfully attenuated the relationship. The inability of self-reported experiences of discrimination to explain the racial inequalities in hypertension may reflect a lack of attention in the study to moderating factors that influence how a person deals with or reacts to perceived discrimination. For example, James [11] suggests that ‘high effort’ coping with psychosocial stressors produces increases in blood pressure, suggesting that the manner in which someone responds to perceived racism moderates its effects. Along similar lines, Chae and colleagues [12] found no relationship between perceptions of racial discrimination and cardiovascular disease in a sample of African American men but discovered that racial discrimination was positively associated with cardiovascular disease among the men who did not subscribe to negative views about Blacks and negatively associated among the men who *did* subscribe to such views. This means that beliefs and perceptions about the relative worth of racial identities in society may also be enmeshed with discrimination as predictors of health. In short, discrimination may engender hypertension among Black Canadians who actively confront the racism, who identify themselves more strongly as Black and/or who believe that the racism is unfair and unjust; unfortunately, this study could not investigate these possibilities.

## Conclusions

Only educational attainment explained some of the high risk of hypertension for the Black Canadians in this sample, throwing the viability of self-reported discrimination and psychosocial stress as explanations for racial inequalities in hypertension in doubt in this context. Conducting subgroup analyses by gender, discerning between real and perceived experiences of discrimination and incorporating consideration of potentially moderating factors such as coping strategy and internalization of racial stereotypes are important issues to address in future Canadian racial inequalities research.

## Competing interests

I have no competing interests regarding this paper.
